# Anti-trypanosome effects of nutritional supplements and vitamin D_3_: in vitro and in vivo efficacy against *Trypanosoma brucei brucei*

**DOI:** 10.1186/s41182-016-0024-7

**Published:** 2016-08-08

**Authors:** Ripa Jamal, Rieko Shimogawara, Ki-ichi Yamamoto, Nobuo Ohta

**Affiliations:** Department of Environmental Parasitology, Graduate School of Medical and Dental Sciences, Tokyo Medical and Dental University, 1-5-45 Yushima, Bunkyo-ku, Tokyo, 113-8519 Japan

**Keywords:** *Trypanosoma brucei brucei*, Nutritional supplement, Vitamin D_3_, African trypanosomiasis

## Abstract

**Background:**

Previous publications suggest that nutritional supplements have anti-trypanosome activity in vitro, although apparent efficacy was not noted in vivo. This study was conducted by experimentally infecting mice with *Trypanosoma brucei brucei* to assess the anti-trypanosome activity of various nutritional supplements with the hope of finding possible application in the treatment of African trypanosomiasis.

**Methods:**

Activities of nutritional supplements were screened in vitro against bloodstream forms of *T. b. brucei*. To evaluate selectivity, we used two mammalian cells, Jurkat cells and Vero cells. The IC_50_ values and selectivity index values were calculated, and supplements with promising efficacy in vitro were selected for further testing in vivo. Mice were infected intraperitoneally with 1 × 10^3^*T. b. brucei*. We observed parameters for disease progression such as parasitemia, red blood cell count, white blood cell count, survivability, and splenomegaly. Morphological profiles after the treatment were analyzed by scanning electron microscopy.

**Results:**

Vitamin D_3_ showed anti-trypanosome efficacies both in vitro and in vivo. It seemed to have suppressive effects on parasitemia, and spleen weight was also significantly lower in vitamin D_3_-treated mice when compared to non-treated control mice. There was, however, no significant prolonged survivability of infected mice treated with vitamin D_3_. Among green tea extracts, polyphenon-60 and epigallocatechin gallate had suppressive effects against *T. b. brucei* in vitro, but in vivo efficacies were marginal.

**Conclusions:**

Treatment with nutritional supplements, vitamin D_3_, and polyphenon-60 seemed to have anti-trypanosome activity in vitro and protective activity to some extent in vivo, respectively, although those supplements themselves did not have curable effects. The exact mechanisms of action are not clear, but the significant efficacy in vitro suggested direct effects of supplements against African trypanosome parasites.

## Background

African trypanosomiasis consisting of human African trypanosomiasis (HAT) and animal African trypanosomiasis (AAT/Nagana) are listed as neglected tropical diseases endemic in sub-Saharan Africa [1, 2]. It is caused by parasitic protist of the order Kinetoplastida and genus *Trypanosoma*. HAT is primarily caused by two subspecies of the protozoan parasite, i.e., *Trypanosoma brucei gambiense* and *Trypanosoma brucei rhodesiense*. In Western and Central African subregions, *T. b. gambiense* is the major causative parasite of the disease, while in Eastern Africa, *T. b. rhodesiense* predominates. A third closely related subspecies, *Trypanosoma brucei brucei*, cannot survive in the human host due to the human serum lytic factor but is responsible for many cases of nagana in cattle in East Africa [3, 4].

Trypanosomes are transmitted by insect vector, tsetse flies (*Glossina* spp.). At the onset of infection, the parasites proliferate in the bloodstream and lymphatic system and after a few weeks, they are able to cross the blood-brain barrier and enter the central nervous system. Once this occurs, patients show a variety of neurological symptoms becoming fatal without proper treatment resulting in coma and ultimately death [5–7]. Therefore, effective treatment at the early stage is crucial. The current drugs available for treatment of trypanosomiasis are less effective with severe side effects. Furthermore, the route of administration of these drugs and their optimal doses should be re-considered to achieve better treatment efficacy [6].

The host immune response system is known to play an important role in the disease progression and is considered to be highly essential for the control of the early phase of parasite replication which may be associated with host resistance to the parasite proliferation [8]. The use of nutritional supplements to aid in host response against protozoan parasites has been studied, some of which revealed anti-trypanosome effects. Ascorbic acid (vitamin C) has been shown to aid in combating the oxidative stress injuries in vital organs of mice infected with *Trypanosoma cruzi* [9]. Epigallocatechin gallate (an analog of green tea extract) administration to mice showed significant levels of decreased parasitism and increased survival rates suggesting that epigallocatechin gallate may be potentially useful for the protection against *T. cruzi* [10]. Zinc supplementation has also shown beneficial activity in the reduction of parasitemia in *T. b. brucei*-infected mice [11]. Considering the situation that no safe and effective drugs are available, treatment regimens using drugs and supplements might be a challenge for the trypanosomiasis control strategy.

In view of these, we postulated that nutritional supplements may have some inhibitory/protective activity against *T. b. brucei*. Thus, we screened various nutritional supplements from different functional groups for their inhibitory effects in vitro and compounds that showed promising results were further assessed in vivo for their protective abilities.

## Methods

### Parasite strains

*T. b. brucei* bloodstream forms of strain GUTat 3.1 and TC221 maintained at Tokyo Medical and Dental University were used for all in vitro and in vivo experimental procedures. Parasites were cultured in Iscove’s modified Dulbecco’s medium (IMDM) (Sigma-Aldrich, St. Louis, MO, USA) supplemented with two mixtures; mixture 1 consisted of 0.1 M HCl, 100 μM hypoxanthine (Sigma-Aldrich), 30 μM thymidine (Sigma-Aldrich), and 40 μM adenosine (WAKO, Osaka, Japan) and mixture 2 consisted of a mixture of 1 mM sodium pyruvate (WAKO), 200 μM l-alanine (WAKO), 100 μM glycine (WAKO), 20 μM l-ornithine monohydrochloride (Tokyo Kasei, Tokyo, Japan), 10 μM l-citrullin (Sigma-Aldrich) and 100 μM 2-mercaptoethanol (WAKO), and 200 ml of distilled water. The adjusted IMDM had a further 10 % fetal bovine serum (FBS), 2 mM l-glutamine (WAKO), and 100 U/ml penicillin-100 μg/ml streptomycin added to it. Parasites were cultured at 37 °C and 5 % CO_2_ humid atmosphere as described previously [12].

### Supplements tested in this study

All supplements used in the experimental protocol are listed in Table 1. Vitamin D_3_ (Cayman Chemical, Ann Arbor, MI, USA), Vitamin C (WAKO), catechin hydrate (Sigma-Aldrich), polyphenon-60 (Sigma-Aldrich), Vitamin E (WAKO), epigallocatechin gallate (Sigma-Aldrich), coenzyme Q_10_ (WAKO), and 5-aminolevulinic acid (WAKO) were tested for their activities against *T. b. brucei*. Vitamin D_3_ was dissolved in 100 % ethanol; the other supplements were dissolved in 100 % dimethyl sulfoxide (DMSO) before use. Suramin (WAKO) and curcumin (WAKO) were used as positive controls.

**Table 1:** Supplements and chemicals selected for in vitro screening of *T. b. brucei*

Chemical name	Structural formula	Molecular weight (g/mol)	Function
Polyphenon-60		290.3	Mainly anti-oxidative effects and also anti-cancer, anti-inflammatory [31]
Catechin hydrate		290.3	Able to impart thermal stability to collagen [32]
Epigallocatechin gallate		458.4	Modulates cognitive function and brain activity [33]
5-Aminolevrinic acid		167.6	Helps in heme synthesis, heme protein design [34]
Coenzyme Q_10_		863.34	Act as an anti-oxidant, needed for basic cellular function [35]
Vitamin C		198.11	An anti-oxidant, required for collagen synthesis and biosynthesis of certain hormones [36]
Vitamin D_3_		384.64	An immunomodulator, supports the brain and nervous system [37]
Vitamin E		430.71	Essential for neurological function [38]
Curcumin		368.38	Exhibits anti-cancer, chemo- preventive, chemo- and radio-sensitization properties [39]

### Efficacy of supplements against *T. b. brucei* in vitro

Effects of the nine supplements against the bloodstream form of *T. b. brucei* GUTat 3.1 were determined using the alamarBlue method (Sigma-Aldrich) as was shown elsewhere [13]. Each well contained 1 × 10^5^ cells in 100 μl medium with supplement concentrations in 1:2 serial dilutions (100~1.5 μM) except for vitamin D_3_. Vitamin D_3_ had a starting concentration of 13 μM and also dissolved in a 1:2 serial dilution. To check the viability of trypanosomes at 24 and 48 h, 10 μl of alamarBlue solution was added to each well at 23 and 47 h of incubation, respectively, followed by further 1 h incubation. Fluorescence was read in FLUOstar OPTIMA (BMG Labtech, Aylesbury, UK; λ_excitation_ = 540 nm; λ_emission_ = 590 nm) and absorbance measured by TriStar LB941 (Belthold, Oak Ridge, TN, USA). We determined selectivity index of tested supplements against trypanosome cells by comparing the efficacies against two mammalian cells, Jurkat cells and Vero cells. Cells were cultured in conditioned medium of Roswell Park Memorial Institute medium-1640 (RPMI-1640) with 10 % FBS or Dulbecco’s modified Eagle’s medium (D-MEM) (WAKO) for 24 h and were exposed to various concentrations of supplements for 48 h. Sensitivity of the mammalian cells was measured using alamarBlue assay, as described previously [13].

### Testing in experimental murine infection model

Female BALB/c mice aged 6 weeks were purchased from CLEA (Tokyo, Japan). Mice were randomly assigned into five mice/group and treated with three test supplements, vitamin D_3_, polyphenon-60, catechin hydrate, positive control (suramin), and a healthy control (infection-free). Sample size for mice was chosen by referring to a previous report [14]. Catechin hydrate and polyphenon-60 dissolved in DMSO were administered at a dose of 50 mg/kg via intraperitoneal route every 2 days in experimental period. Vitamin D_3_ was dissolved in ethanol and administered at a dose of 250 ng/kg by subcutaneous injection. After that, mice were infected with 1 × 10^3^*T. b. brucei* TC221 via the peritoneum. Suramin was used at a dose of 30 mg/kg, while phosphate-buffered saline (PBS) (pH 7.4) was used as a positive control. The parameters used to assess the disease progression were body weight, level of parasitemia, total red blood cell count (RBC count), total white blood cell count (WBC count), survivability, and splenomegaly. All parameters were measured independently by two researchers to reduce bias and for comparison. In cases of discordant results, the parameters were measured again for confirmation and consistency. Animal study was done in strict accordance with the guidelines approved by the Committee of Animal Ethics of Tokyo Medical and Dental University (0160295A, 2015, and 0170274A, 2016).

### Scanning electron microscopy

*T. b. brucei* GUTat 3.1 treated with supplements in vitro were fixed in 3.5 % glutaraldehyde placed in a fridge at 4 °C for overnight fixation. The next day, it was washed with PBS. Small droplets containing parasites were put on poly-lysine coated slides. Samples on slide glass were then stained in 1 % osmium tetraoxide (OsO_4_) and dehydrated in a series of graded ethanol concentrations for 10 min each. They were dried in a critical point dryer (Hitachi, Tokyo, Japan) using liquid carbon dioxide. The samples were then sputter coated with platinum in an ion sputter coater (Hitachi). Digital images were collected using a scanning electron microscope (S-4500, Hitachi) operating at 10 kV at 500 to 40,000 times magnification.

### Data analysis

Data were presented as mean values with standard deviation. The data were subjected to analysis of variance using ANOVA. Means were considered significant at 95 % confidence interval with a *p* value <0.05.

## Results

### In vitro effects of the supplements against *T. b. brucei*

Nutritional supplements with possible anti-trypanosome efficacy were assayed for inhibitory activity against *T. b. brucei*. In the in vitro assay, polyphenon-60, vitamin D_3_, and epigallocatechin gallate showed relatively low IC_50_ values of 16.9, 4.58, and 8.40 μM, respectively, which were, however, less effective than that of suramin (1.18 μM) (Table 2). From the results obtained by testing those supplements against mammalian cells, the selectivity index of vitamin D_3_ was good enough, but other supplements had medium or low selectivity index values (Table 2). Based on these in vitro results, we selected vitamin D_3_ and polyphenon-60 to observe the effects in vivo*.*

**Table 2:** Screening IC_50_ values of nutritional supplements against *T. b. brucei* and mammalian cells in vitro

Supplement	*T. b brucei* ^a^	Mammalian cells			
		Jurkat cells	SI	Vero cells	SI
5-Aminolevrinic acid	51.3 μM	>100 μM	>1.95	>100 μM	>1.95
Catechin hydrate	53.6	>100	>1.86	>100	>1.86
Coenzyme Q_10_	>100	>100	ND	>100	ND
Epigallocatechin gallate	8.40	35.7	4.25	39.8	4.74
Polyphenon-60	16.9	26.4	1.56	41.2	2.44
Vitamin C	58.6	>100	>1.70	>100	>1.70
Vitamin D_3_	4.58	>100	>21.8	>100	>21.8
Vitamin E	28.6	>100	>3.49	>100	>3.49
Curcumin	6.05	34.3	5.66	90.4	>1.70
Suramin	1.18	>100	>84.8	>100	>84.8

*ND* not determined, *SI* selectivity index

^a^ *T. b. brucei* GUTat 3.1 strain was used

### In vivo observation of anti-trypanosome activity of supplements

**Fig. 1:**
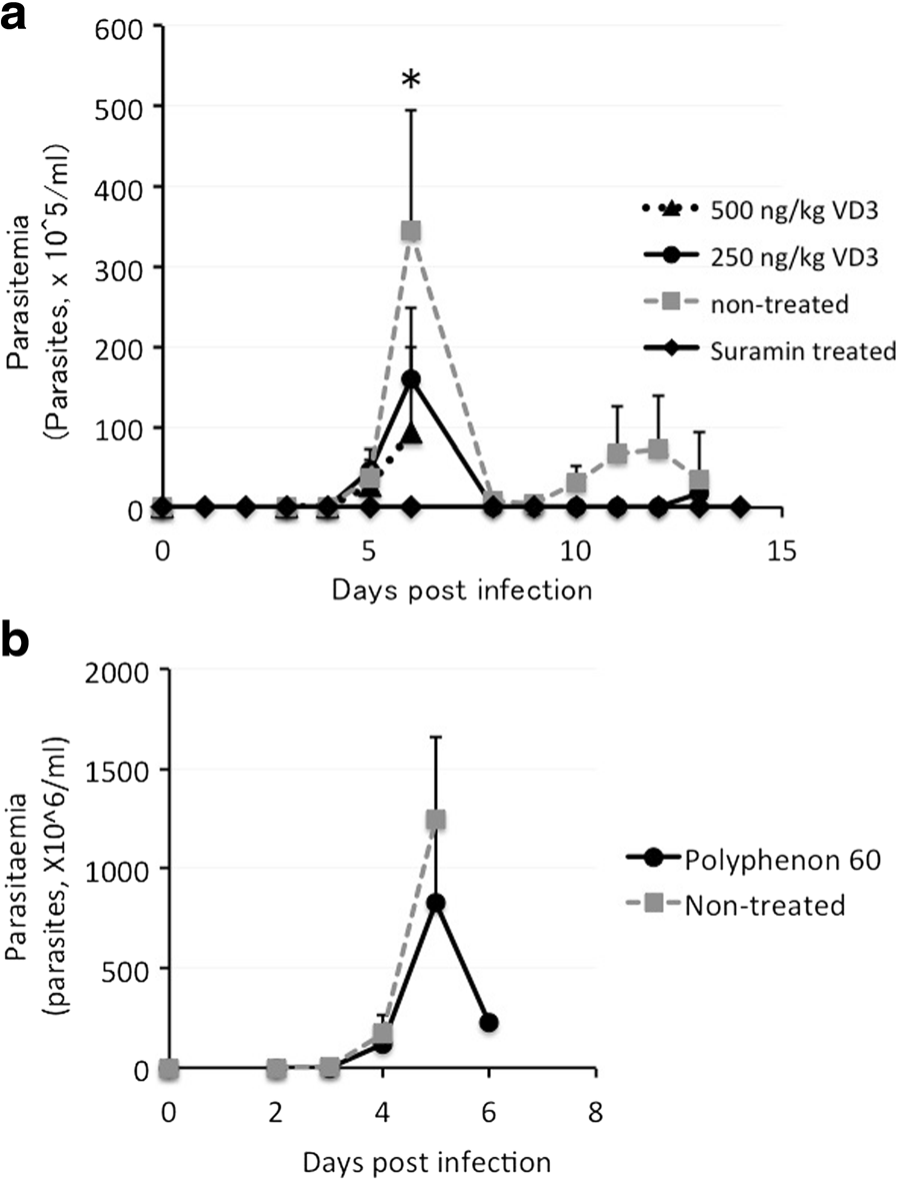
Reductions of parasitemia level of supplement-treated mice. **a** Parasitemia levels in mice infected with *T. b. brucei* GUTat 3.1 and treated with 500 ng/kg vitamin D_3_ and 250 ng/kg vitaminD_3_. *Solid triangles* (▲) *with a dotted line* show parasitemia of 500 ng/kg vitamin D_3_-treated group. *Solid circles* (●) *with a solid line*, *closed squares* (■) *with broken line*, and *solid diamonds* (◆) *with a solid line* show 250 ng/kg vitamin D_3_-treated group, non-treated control group, and suramin-treated group, respectively. **b** Parasitemia levels in mice infected with *T. b. brucei* and treated with 50 mg/kg polyphenon-60. *Solid circles* (●) *with solid line* show survival rates of polyphenon-60-treated group, and *solid squares* (■) *with a broken line* show those of non-treated control group. Vitamin D_3_-inhibited level of parasitemia was significantly lower than that of control-infected mice (*P* < 0.05). Polyphenon-60-treated group also showed inhibition, but it was not statistically significant (*P* = 0.056)

**Fig. 2:**
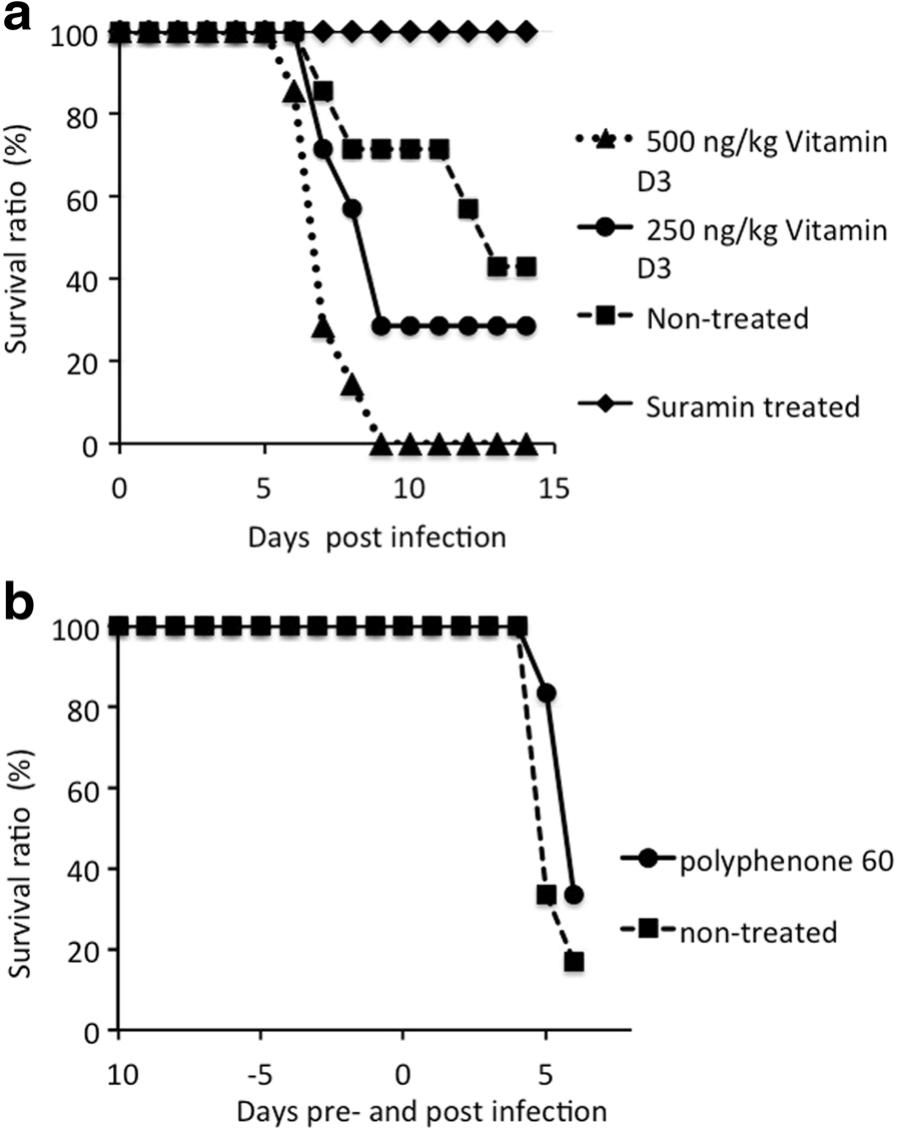
Survivability of trypanosome-infected mice treated with supplements. **a** Survival rates of vitamin D_3_-treated mice infected with *T. b. brucei* GUTat3.1. *Solid triangles* (▲) *with a dotted line* show survival rate of 500 ng/kg vitamin D_3_-treated group. *Solid circles* (●) *with a solid line*, *closed squares* (■) *with a broken line*, and *solid diamonds* (◆) *with dotted line* show 250 ng/kg vitamin D_3_-treated group, non-treated control group, and suramin-treated group, respectively. **b** Survival rates of polyphenon-60-treated mice infected with *T. b. brucei* GUTat 3.1. *Solid circles* (●) *with solid line* show survival rates of polyphenon-60-treated group and *solid squares* (■) *with a broken line* show those of non-treated control group. Polyphenon-60-treated mice showed slight prolongation of survivability, but those were not statistically significant difference in comparison with that of non-treated control

**Fig. 3:**
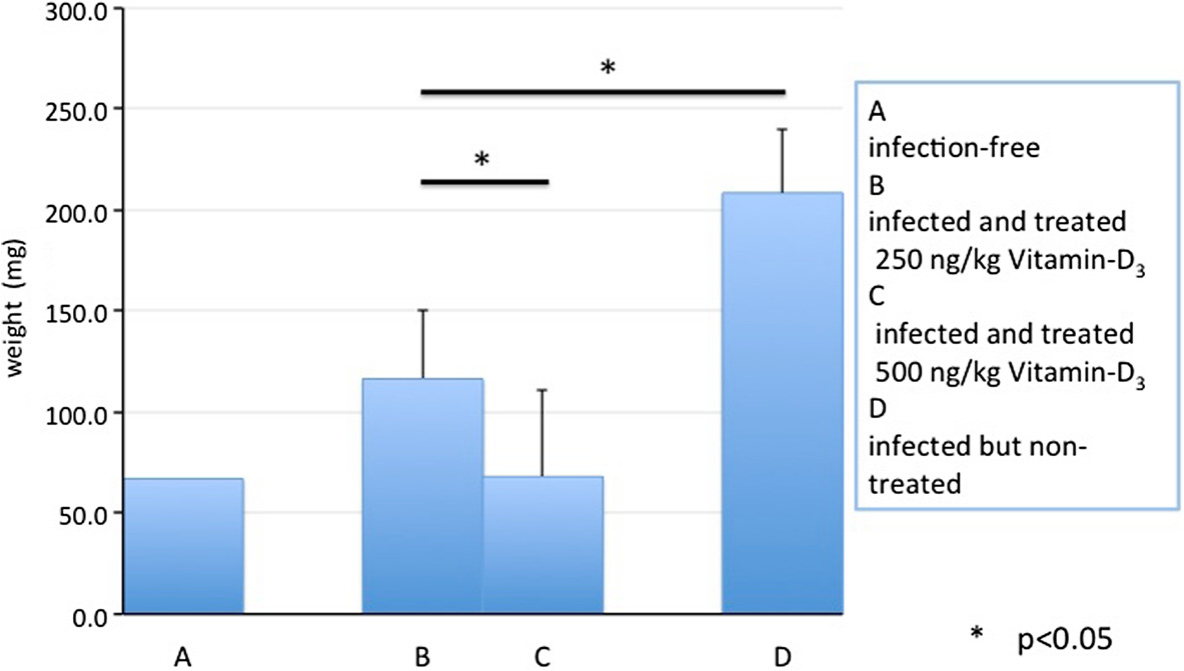
Comparing spleen weight treated or non-treated with vitamin D_3_. Spleen weight was compared among four groups of mice: *a* infection-free, *b* 250 ng/kg vitamin D_3_-treated, *c* 500 ng/kg vitamin D_3_-treated, and *d* non-treated control, while *b–d* were infected. There was a significant reduction in spleen weight in vitamin-D_3_-treated groups compared with treatment-free control mice

Selected nutritional supplements were assessed for five parameters in vivo. During the 10 days before trypanosome infection, treatments with polyphenon-60 made no significant changes in body weight and RBC and WBC count, suggesting no apparent negative effects for mice. After trypanosome infection, efficacies of supplement treatment on three parameters assessed are summarized in Table 3. In brief, there was no detectable difference in body weight but continuous administration of vitamin D_3_ may cause statistically significant differences in body weight. When we compared results of parameters of RBC count and WBC count with infection-free control mice, RBC count recovered by vitamin D_3_ treatment, polyphenon-60, and suramin, indicated that treated mice recovered from anemic conditions induced by infection with *T. b. brucei.* On the other hand, WBC count significantly reduced in infected groups (*p* < 0.05), and treatment with supplements (vitamin D_3_ and polyphenon-60) and suramin resulted in more profound leukopenic conditions. Vitamin D_3_ provided significant decrease in parasitemia compared with that of non-treated control mice (Fig. 1) and seemed to be dose-dependent (Fig. 1). Polyphenon-60 showed suppression of parasites, but it showed marginal statistical difference (*p* = 0.056). Although survivability of mice treated with polyphenon-60 was slightly prolonged, there was no statistical significance when compared with that of non-treated control (Fig. 2). Suramin-treated group showed 100 % survivability during the period of our observation. Splenomegaly in vitamin D_3_-treated mice was significantly suppressed in comparison with non-treated mice in a dose-dependent manner (*p* < 0.05) (Fig. 3). Considering that splenomegaly is one of the signs of disease progression of African trypanosomiasis, treatment of vitamin D_3_ might have had some protective effect.

**Table 3:** Body weight, RBC, and WBC levels in mice after pre- and post-infection stratified according to supplement administration

Parameters			*T. b. brucei* infection (+) and treatment with			
		Infection-free	No treatment	Vitamin D_3_	Polyphenon-60	Suramin
Body weight (g)	Pre-infection^b^	20.0 ± 1.05	22.4 ± 1.32		22.2 ± 0.89	
	Post-infection^c^		21.6 ± 0.94	20.4 ± 1.46	18.6 ± 0.96	20.3 ± 0.56
RBC (×10^6^/ml)	Pre-infection^b^	8.30 ± 0.62	8.45 ± 0.37		11.2 ± 0.22	
	Post-infection^c^		6.42 ± 0.94^a^	10.9 ± 0.11	8.10 ± 0.23	9.90 ± 0.97
WBC (×10^3^/ml)	Pre-infection^b^	5.90 ± 0.11	4.40 ± 0.14		4.17 ± 0.56	
	Post-infection^c^		4.60 ± 0.15^a^	2.20 ± 0.44^a^	3.50 ± 0.16^a^	3.70 ± 0.56^a^

^a^Statistically significant differences between the four groups and infection-free group are shown after analysis of variance (ANOVA). *p* value is less than 0.05 to the chosen significance level

^b^Pre-infection: on day 4 of polyphenon-60 treatment but not yet infected with *T. b brucei* GUTat 3.1 strain

^c^Post-infection: 7 days after infection

### Scanning electron microscopy assessment of treated trypanosomes

**Fig. 4:**
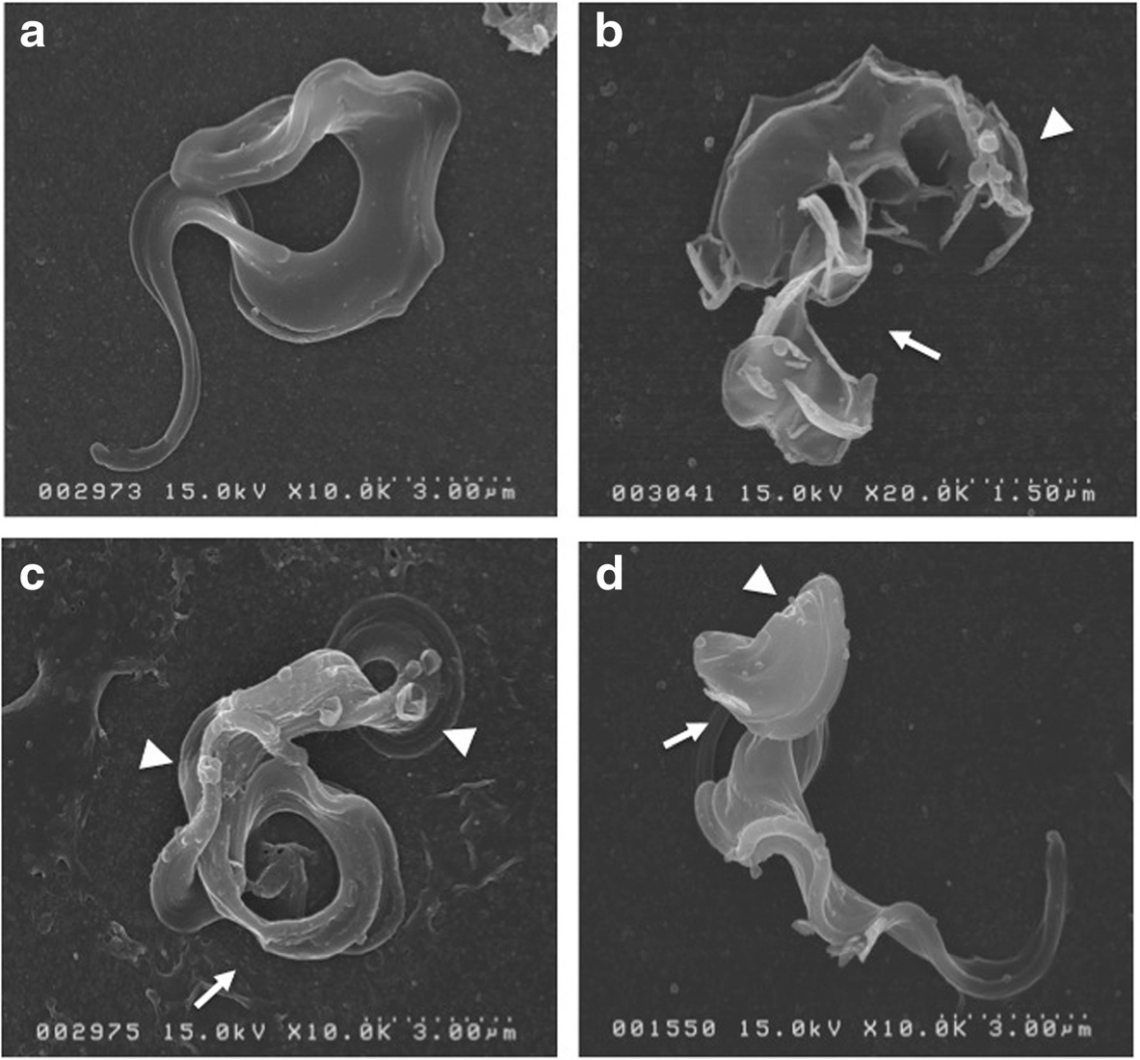
SEM morphology assessment of *T. b. brucei* GUTat 3.1 strains cultured in vitro with various treatment protocols. **a** Non-treated control, **b** suramin-treated, **c** vitamin D_3_, and **d** epigallocatechin gallate (an analog of green tea extract)-treated. Suramin-treated parasites showed detachment of the flagella from the body and surface damage. In cases of vitamin D_3_ or polyphenon-60 treatment, slight changes of the cell surface showing irregular knot-formation, there was apparent damage in the flagella structure. *Arrow head* showed flagella damage, and *Arrow* showed damaged cell membrane

Morphological profiles of *T. b. brucei* treated with vitamin D_3_, were assessed by SEM (Fig. 4). Suramin-treated parasites showed apparent changes such as displacement of flagellum from the body surface, pouching, and other related damage to the cell surface. In comparison with non-treated trypanosomes, we observed slightly jagged surface of vitamin D_3_-treated parasites, damaged flagellum was observed in the suramin treatment group.

## Discussion

Human African trypanosomiasis is still a disease of tremendous public health and economic importance, but the major challenge in the control is the development of safe and effective drugs available for treatment [15]. In parallel with therapeutic drugs, various nutritional supplements have been tested for inhibitory effects, if not therapeutic, against African trypanosomiasis [10, 11, 16]. In our study, we examined the possible efficacy of available nutritional supplements in enhancing host defense response to suppress parasite growth and disease progression.

Results obtained here suggested that vitamin D_3_ and polyphenon-60 showed anti-trypanosome activity both in vitro and in vivo, while catechin hydrate showed only a faint effect in vivo (data not shown). Vitamin D_3_ showed a parasite growth inhibition in vitro with a good selectivity index. Although vitamin D_3_ failed to show curable effects in vivo, significant parasite growth inhibition was observed in vivo, and also splenomegaly during the disease course was suppressed. In this sense, vitamin D_3_ cannot be a therapeutic drug for African trypanosomiasis in the protocol tested here which is severely dose dependent, but it might be expected to enhance efficacy of therapeutic drugs. Previous researchers have postulated that vitamin D reduced the risk of certain infections through multiple mechanisms [17, 18]. It has been shown that vitamin D boosts innate immunity by modulating production of anti-microbial peptides (AMPs) and cytokine response [19, 20]. For an experimental *T. cruzi* infection, vitamin D treatment favored the hosts be able to overcome acute phase of the disease and to prolong survivability in the chronic phase [18].

When efficacy of vitamin D was discussed, vitamin D was likely to have an effect indirectly to the pathogen. For instance, vitamin D exerts an immunomodulatory effect through complex interactions with vitamin D receptors (VDR) [21]. VDR is a member of the superfamily of nuclear hormone receptors expressed on immune cells in humans [22, 23]. Circulating vitamin D levels has a direct influence on macrophages, increasing their oxidative potential [24]. There was a report showing that vitamin D_3_ facilitates neutrophil motility and phagocytic function [25]. All those seem to show that the efficacy of vitamin D was indirect to the pathogen, but rather immune cells were activated and subsequently suppressed parasite growth in vivo. In this sense, our observation suggests that vitamin D_3_ could have direct effect(s) against *T. b. brucei*, since treatment of vitamin D_3_ in vitro in the absence of host immune cells also has detectable inhibitory effects.

Polyphenon-60 is a derivative of green tea extracts. It has been suggested that green tea extracts including catechin seem to have inhibitory effects against *T. b brucei* in vitro. Polyphenon-60 also showed significant inhibitory effect against parasite proliferation in vitro. Furthermore, slight prolongation of survivability was observed in infected mice treated with polyphenon-60, although it was not statistically significant. Administration of tea extracts has also been previously shown to prevent reduction in albumin concentration during *T. b. brucei* infection in mice, thereby suggesting decreased inflammation due to the trypanosome parasite [26]. Polyphenon-60 has extensive protein-denaturing characteristics as shown against influenza virus. Alternatively, polyphenon-60 is a supplement with anti-oxidative effects and immune-enhancing effects as has been already reported [27]. It is possible to speculate that those functions contributed to anti-trypanosome activities of polyphenon-60 in our study. It is again likely that polyphenon-60 could support and/or enhance the efficacy of anti-trypanosome drugs.

Nutritional supplements are not necessarily safe in daily use. Previous research had shown that vitamin D_3_ treatment produced calcinosis lesions in the myocardium, coronary and kidney arteries, and may have induced mice death [28]. In our study, several mice died during the treatment of vitamin D_3_ even in the situation of relatively low parasitemia level (Fig. 1) while using high dose of vitamin D_3_ (500 ng/kg). Although we did not study the possible histopathological changes due to vitamin D_3_ treatment, similar phenomena were observed by one of the authors in the murine experimental malaria (KY, manuscript in preparation). Probably, *T. b. brucei* infection could produce acute and chronic fatal conditions just like splenomegaly to the host [29]. On the other hand, polyphenon-60 has been shown to have toxic effects when administered in high doses to mice, where they induce reactive oxygen species formation, and affect mitochondrial membrane potential thereby causing death [30]. In the murine experimental malaria, high doses of polyphenon-60 administration caused significant toxicity and mortality in the infected mice (KY, unpublished data). Such information strongly suggests that supplement treatment should be under strict control to avoid negative effects. More detailed studies are needed to establish the optimal protocol for safe and effective treatment of supplements with anti-trypanosome activities.

## Conclusions

We observed inhibitory effects of vitamin D_3_ against *T. b. brucei* in vitro and possibly in vivo. We were not able to observe curable effects for this supplement in vivo; several parameters of disease progression were improved by the treatment with this supplement. The results obtained suggest that treatment with nutritional supplements may possibly have protective/prophylactic roles; we therefore recommend that vitamin D_3_ and green tea extracts could be included in the treatment protocols. In addition, further test should be conducted to confirm the efficacy of dietary supplements as treatment regimens in trypanosome infections.

## Abbreviations

AAT, animal African trypanosomiasis; D-MEM, Dulbecco’s modified Eagle’s medium; DMSO, dimethyl sulfoxide; FBS, fetal bovine serum; HAT, human African trypanosomiasis; IC_50_, half maximal (50 %) inhibitory concentration (growth rate); IMDM, Iscove’s modified Dulbecco’s medium; PBS, phosphate-buffered saline; RBC, red blood cell; RPMI-1640, Roswell Park Memorial Institute medium-1640; SEM, scanning electron microscopy; *T. cruzi*, *Trypanosoma cruzi*; *T. b. brucei*, *Trypanosoma brucei brucei*; VDR, vitamin D receptors; WBC, white blood cell
